# Genetic Variants in *PVRL2-TOMM40-APOE* Region Are Associated with Human Longevity in a Han Chinese Population

**DOI:** 10.1371/journal.pone.0099580

**Published:** 2014-06-12

**Authors:** Fang Lu, Huaijin Guan, Bo Gong, Xiaoqi Liu, Rongrong Zhu, Yong Wang, Jingjing Qian, Tianqiu Zhou, Xiaoyan Lan, Pu Wang, Ying Lin, Shi Ma, He Lin, Xiong Zhu, Rong Chen, Xianjun Zhu, Yi Shi, Zhenglin Yang

**Affiliations:** 1 Sichuan Key Laboratory for Disease Gene Study, Sichuan Academy of Medical Sciences & Sichuan Provincial People's Hospital, Sichuan, China; 2 Eye Institute, Affiliated Hospital of Nantong University, Nantong, Jiangsu, China; 3 Sichuan Translational Medicine Research Hospital, Chinese Academy of Sciences, Chengdu, Sichuan, China; 4 School of Medicine, University of Electronic Science and Technology of China, Chengdu, Sichuan, China; Central China Normal University, China

## Abstract

**Purpose:**

Human longevity results from a number of factors, including genetic background, favorable environmental, social factors and chance. In this study, we aimed to elucidate the association of human longevity with genetic variations in several major candidate genes in a Han Chinese population.

**Methods:**

A case-control association study of 1015 long-lived individuals (aged 90 years or older) and 1725 younger controls (30–70 years old) was undertaken. Rs2075650 in *TOMM40* was firstly genotyped using the ABI SNaPshot method in an initial cohort consisted of 597 unrelated long-lived individuals and 1275 younger controls enrolled from Sichuan. Secondly, eighteen tag single-nucleotide polymorphisms (SNPs) in the *PVRL2-TOMM40-APOE* locus were genotyped for extensive study in the same cohort. Finally, 5 associated SNPs were genotyped in a replication cohort including 418 older individuals and 450 younger controls. The genotype and allele frequencies were evaluated using the χ^2^ tests. The linkage disequilibrium (LD) block structure was examined using the program Haploview.

**Results:**

The case-control study of rs2075650 in *TOMM40* showed significant difference in allele frequencies between cases and controls (*P* = 0.006) in an initial study. Of the 18 SNPs genotyped, rs405509 in *APOE* and another three SNPs (rs12978931, rs519825 and rs395908) in the *PVRL2* gene also showed significant association with human longevity in extensive study in the same cohort. Rs2075650 in *TOMM40*, rs405509 in *APOE* and rs519825 in *PVRL2* showed a significant association with human longevity in a replication cohort.

**Conclusion:**

These results suggested that *PVRL2*, *TOMM40* and *APOE* might be associated with human longevity. However, further research is needed to identify the causal variants and determine which of these genes are involved in the progress of human longevity.

## Introduction

Human longevity is considered a multi-factorial phenotype [1]. Worldwide human populations have shown an increase in mean life expectancy in the past two centuries [2], [3]. This is mainly due to environmental factors such as improved hygiene, nutrition and health care. The large variation in healthy lifespan among the elderly has prompted research into the determinants of aging and lifespan regulation. Longevity and healthy aging associated genetic research may provide further insights into the mechanisms of aging [4]–[7]. The genetic component has been shown to become stronger with increasing age of the individuals [8], [9]. The genetic contribution to human lifespan variation was estimated at 25–30% in twin studies [8], [10], [11]. The most prominent genetic influence is observed in families in which the capacity to attain a long lifespan clusters [12]–[14]. Epidemiological data indicate the presence of a strong familiar component of longevity that is largely determined by genetics and progeric syndromes of accelerated aging have known genetic causes [15]. Very long life, to beyond the age of 90 years, appears to have an even stronger genetic basis [16], which explains why centenarians and near-centenarians tend to cluster in families. Exceptional longevity can be reached with a low degree of age-related disability [17], [18], raising the question whether protective mechanisms against disease exist in long-lived subjects.

People who survived with long life (centenarians, octogenarians and nonagenarians) are characterized by marked delay or escape from age-related diseases, therefore analysis of genes that modulate susceptibility to age related diseases in these populations may provide insights into the human longevity [4]. Candidate genes for longevity encode proteins engaged in different biological processes including lipoprotein metabolism and inflammatory processes [1]. Several genes by far have repeatedly been associated with human longevity, such as *APOE*, *FOXO3A* and *AKT1* [19]–[26].

Therefore, the present study was carried out to replicate and extend previous findings by testing the association of candidate genes previously reported to contain lifespan associated polymorphisms (including rs2075650 in *TOMM40* and rs405509 in *APOE*), so that we could investigate the association of human longevity with genetic variations in major candidate genes in the Han Chinese population.

## Materials and Methods

### Subjects

In this study, the samples comprised of 2740 participants including 1015 individuals (mean age: 93.1±5.7 years, range: 90–105 years old) and 1725 younger controls (mean age: 57.3±10.3 years, range: 30–70 years old) (**Table 1**). They composed of initial cohort (597 older individuals and 1275 younger controls) from Sichuan Province and replication cohort (418 older individuals and 450 younger controls) from Nantong city in Jiangsu Province. They were all of Han Chinese ethnicity. This study was approved by the Institutional Review Boards of the Sichuan Academy of Medical Sciences & Sichuan Provincial People's Hospital. Written informed consents were obtained from all subjects prior to the studies.

**Table 1:** Samples used for analysis.

Characteristics of the genotyped samples used for analysis	Cases	Controls
**Initial cohort (Rs2075650 and another 18 SNPs)**	597	1275
**Mean age (year)** *	92.9±6.5	55.3±11.3
**Age range (year)**	90–105	30–70
**Replication cohort (5 SNPs)**	418	450
**Mean age (year)** *	93.3±5.8	58.3±9.7
**Age range (year)**	90–105	30–70

*The age when the cases and controls were recruited. ±: standard deviation;

### Selection of candidate genes and SNPs

Rs2075650 in *TOMM40* and another 18 tag SNPs in the *PVRL2-TOMM40-APOE* locus were chosen based on comprehensive literature/data base searches in different candidate longevity genes. Tag SNPs were identified according to the HapMap Phase II+III (Feb. 2009) of CHB database (Han Chinese in Beijing, China) and analyzed by using the Haploview software (version 4.2).

### Extraction and quantification of genomic DNA

Venous blood from each subject was drawn and collected in an EDTA tube. Genomic DNA was extracted from peripheral blood with a QIAamp DNA Blood Maxi Kit (Qiagen), and was fluorometrically quantified with Quant-iT PicoGreen reagent (Invitrogen) according to the manufacturer's protocol.

### SNP genotyping

To determine if any polymorphic variant is associated with human longevity between the long-lived individuals and younger controls, we firstly carried out the case-control study of rs2075650 in *TOMM40* in an initial cohort from Sichuan (**Table 1**) using the ABI SNaPshot method. Secondly, 18 tag SNPs in and around the *TOMM40* and *APOE* locus covering the common genetic variation were followed to be genotyped for extensive study in the initial cohort. Finally, associated SNPs from extensive study were genotyped in a replication cohort.

SNP analysis was performed on the ABI 3130 Genetic Analyzer (Applied Biosystems, CA, USA). In brief, the polymerase chain reactions (10 µL final volume) contained 50 ng of genomic DNA, 1 µL of each primer (10 pmol/µL), 1 µL of 10 buffer (Takara Bio Inc., Shiga, Japan), 0.8 µL of deoxyribonucleotide triphosphates (2 mmol/L; Takara Bio Inc.), 0.4 µL MgCl2 (2.5 mmol/L; Takara Bio Inc.), and 0.1 µL of ExTaq polymerase (5 U/µL; Takara Bio Inc.). The product was then processed according to the ABI SNaPshot protocol using primers designed for fluorescent dideoxy nucleotide termination. The SNPs reported in this manuscript have a genotyping success rate of >98% and accuracy as judged by random re-genotyping of 5% of the samples in the cohort by sequencing analysis.

### Statistical analysis

A standard χ^2^ test with a 1-degree-of-freedom (df) was used to assess the Hardy-Weinberg equilibrium (HWE) and the differences of allele frequencies for each SNP between the case and control group. Odds ratios (ORs) with 95 percent confidence intervals (CIs) were assessed for the risk allele of each SNP based on a multiplicative model. For the genotypes, we tested a series of genetic models including additive, dominant/recessive for the SNPs with a p value of <0.05 of allelic, trend test by using unconditional logistic regression with adjustment for gender. For multiple correction, the P values were corrected as combined P*5. All statistical analyses were performed by using the software SPSS 15.0 (SPSS Inc., Chicago, IL). The linkage disequilibrium (LD) block structure was examined using the program Haploview (version 4.2, Broad Institute, Cambridge, MA) [27]. The D' values and *r* ^2^ values for all pairs of SNPs were calculated, and the haplotype blocks were estimated using the program haploview.

### Conditional Analysis of *PVRL2-TOMM40-APOE* locus

Conditional analyses of the *PVRL2-TOMM40-APOE* locus were completed at rs12978931 (*PVRL2*), rs519825 (*PVRL2*), rs395908 (*PVRL2*), rs2075650 (*TOMM40*) and rs405509 (*APOE*) in long-lived cases and younger controls. The allelic dosage of each SNP was individually included as an additional covariate in the logistic regression model. Starting with the major allele of one conditional SNP, we performed the likelihood ratio test for independence of the other SNPs to determine the significance of the difference between the alternated SNP model versus unalternated model and see the independent effect on risk of human longevity.

## Results

### Case-control study of rs2075650 in *TOMM40* in an initial cohort

Rs2075650 in *TOMM40* was firstly genotyped in an initial cohort consisted of 597 unrelated long-lived individuals and 1275 younger controls enrolled from Sichuan. The case-control study of rs2075650 in *TOMM40* showed significant difference in allele frequencies (The MAF = 0.070 for cases and MAF = 0.098 for controls, OR = 1.430(95% CI 1.10–1.85), *P* = 0.006) between cases and controls in initial study. SNP rs2075650 is located in the intron of the *TOMM40* gene at chromosome 19q13.32 but it is a strong proxy of the SNPs that define the *APOE* alleles [21], and has been shown a significant association longevity [14], [21], [23], [28].

### Allelic association study of SNPs in and around *TOMM40* in the initial cohort

**Figure 1:**
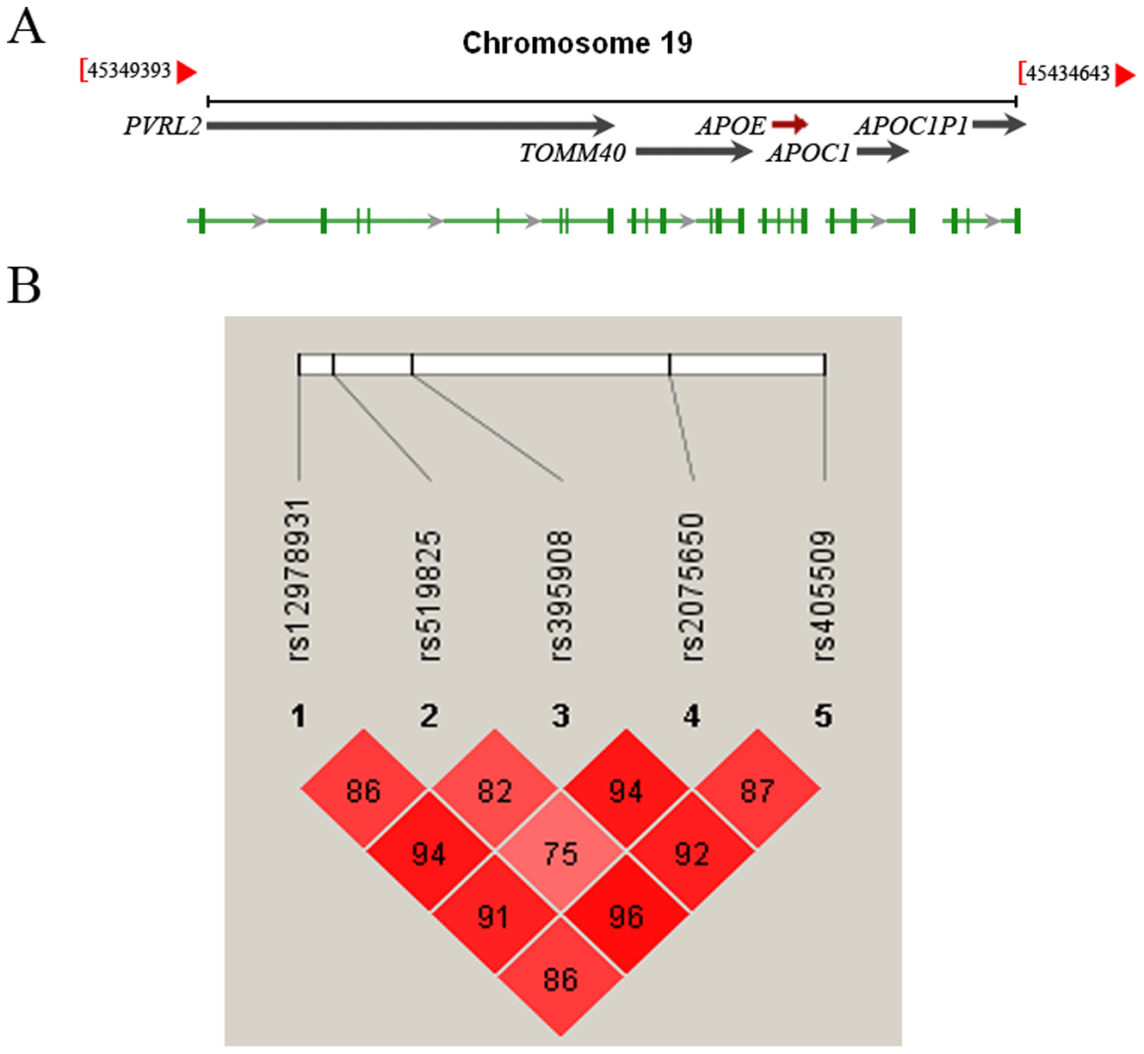
Five SNPs in the genomic region of *PVRL2-TOMM40-APOE* locus. **A**. Genomic region of *PVRL2-TOMM40-APOE* locus associated with human longevity, horizontal arrows indicate the transcriptional orientations of individual genes. **B**. Pairwise LD among five SNPs in and surrounding the *TOMM40* gene in the replication study. The LD spans the region including the *PVRL2, TOMM40* and *APOE*, a distance of 45 kb. Linkage disequilibrium was measured by the D′ statistic using the data from all subjects. A D′ value of 100 indicates a complete LD between 2 markers, and a D′ value of 0 indicates a complete linkage equilibrium. Haploview version 4.2 software was used for the analysis.

Eeighteen tag SNPs in and around the *TOMM40* and *APOE* locus covering the common genetic variation recurrently regarded as candidates for human longevity were genotyped in the initial cohort for extensive study. The *TOMM40* gene located next to *APOE* locus, which is a very important genetic factor influencing longevity [1], [23], [25], as reported as a recent GWAS in Dutch Leiden Longevity Study independently confirmed the *APOE* longevity association [21]. Therefore, we conducted SNP genotyping in and around the *TOMM40* gene to identify novel SNPs associated with human longevity in Chinese population. 18 SNPs, spanning about 45 kb (45.365–45.410 Mb), were selected from a genomic region including four genes (**Figure 1A**) and genotyped for extensive study in the same cohort (**Table 2**). Among them, rs405509 in *APOE* and three SNPs (rs12978931, rs519825 and rs395908) in *PVRL2* showed significant association with human longevity (OR = 1.180(95% CI 1.02–1.37), *P* = 0.027 for rs405509; OR = 1.152 (95% CI 1.00–1.32), *P* = 0.0445 for rs12978931; OR = 0.805(95% CI 0.65–1.00), *P* = 0.048 for rs395908; OR = 1.283(95% CI 1.05–1.57), *P* = 0.01577 for rs519825), whereas another 14 SNPs did not show significant differences between cases and controls in the cohort (**Table 2**). In order to not miss the real association SNPs, we have chosen these five SNPs with P value less than 0.05 but not 0.0026 (multiple correction, 0.05/19 = 0.0026) to the replication study.

**Table 2:** Extensive study for the association of SNPs in the vicinity of *TOMM40-APOE* region.

SNP ID	Position (bp)^a^	Gene	Allele^b^	Genotype counts^e^		minor allele frequency		OR (95% CI)	Allelic *P*
				Cases	Controls	Cases	Controls		
rs1871046	50043777	*PVRL2*	G/A	19/204/374	45/434/796	0.203	0.205	0.983(0.83–1.17)	0.843
rs12978931	50055540	*PVRL2*	G/A	151/283/158	271/628/376	0.494	0.459	1.152 (1.00–1.32)	0.0445
rs519825	50058619	*PVRL2*	G/A	14/143/440	18/258/999	0.143	0.115	1.283(1.05–1.57)	0.01577
rs419010	50060160	*PVRL2*	A/G	261/253/82	489/607/179	0.350	0.378	1.131(0.98–1.31)	0.091
rs4803766	50063008	*PVRL2*	A/G	72/279/246	159/600/514	0.354	0.361	0.972(0.84–1.12)	0.708
rs11879589	50065116	*PVRL2*	A/G	0/21/576	0/27/1248	0.018	0.011	1.673(0.94–2.97)	0.07601
rs395908	50065405	*PVRL2*	T/C	463/121/13	1026/239/10	0.123	0.102	0.805(0.65–1.00)	0.048
rs519113	50068124	*PVRL2*	C/G	8/109/480	23/246/1006	0.105	0.115	0.904(0.72–1.13)	0.3734
rs6857	50084094	*PVRL2*	A/G	0/55/542	0/130/1145	0.046	0.051	0.899(0.65–1.24)	0.518
rs10119	50098513	*TOMM40*	T/C	593/4/0	1267/8/0	0.003	0.003	0.936(0.28–3.12)	0.914
rs449647	50100404	APOE	A/T	21/203/368	53/421/786	0.207	0.209	1.013(0.85–1.20)	0.767
rs405509	50100676	APOE	C/A	63/277/257	112/545/618	0.338	0.302	1.180(1.02–1.37)	0.027
rs440446	50101007	APOE	G/C	23/184/380	49/433/789	0.196	0.209	1.083(0.912–1.289)	0.509
rs769448	50101419	APOE	T/C	0/39/554	0/91/1183	0.033	0.036	1.089(0.744–1.595)	0.660
rs769449	50101842	APOE	A/G	4/109/484	6/213/1051	0.098	0.089	0.895(0.707–1.131)	0.620
rs7412	50103919	APOE	T/C	9/112/470	21/273/970	0.110	0.125	1.152(0.927–1.431)	0.405
rs429358	50103781	*APOE*	C/T	7/90/491	12/230/1033	0.088	0.10	0.877(0.69–1.11)	0.2821
rs4420638	50114786	*APOC1*	G/A	3/104/489	8/266/1011	0.092	0.107	0.851(0.67–1.07)	0.176

a Genomic positions are according to NCBI build 36;

b Minor allele/major allele;

c The genotype counts are presented as homozygote/heterozygote/wildtype;

### Replication study of the five associated SNPs in a replication cohort

Furthermore, the five associated SNPs based on extensive study including three SNPs in the *PVRL2* gene, rs2075650 in *TOMM40* and rs405509 in *APOE* were genotyped in a replication cohort (**Table 3**). Our results showed the three SNPs (rs519825 in *PVRL2*, rs2075650 in *TOMM40* and rs405509 in *APOE*) are significantly associated with human longevity in the replication cohort (allelic *P* = 0.022 for rs519825, *P* = 0.042 for rs405509 and *P* = 0.0030 for rs2075650) (**Table 3**). Combined *P* values of the three SNPs are 0.00082, 0.0006 and 0.002, respectively; corrected *P* values of them are 0.04, 0.03 and 0.01.

**Table 3:** Replication study for the association of the associated SNPs in a replication cohort.

SNP ID	Position (bp)^a^	Gene	Allele^b^	Genotype counts^c^		minor allele frequency		OR (95% CI)	Allelic *P* d	Combined cohort^e^	Corrected*P* f
				Cases	Controls	Cases	Controls				
rs12978931	50055540	*PVRL2*	G/A	105/197/113	96/238/110	0.490	0.484	1.025(0.85–1.24)	0.800	0.053	0.265
rs519825	50058619	*PVRL2*	G/A	10/103/301	8/84/356	0.149	0.112	1.389(1.05–1.84)	0.022	0.00082	0.004
rs395908	50065405	*PVRL2*	T/C	322/79/8	358/84/6	0.114	0.107	0.928(0.69–1.25)	0.628	0.056	0.28
rs2075650	50087459	*TOMM40*	G/A	366/45/4	362/81/6	0.064	0.104	1.693(1.19–2.41)	0.0030	0.0006	0.003
rs405509	50100676	*APOE*	C/A	44/196/171	37/195/217	0.345	0.300	1.234(1.00–1.51)	0.042	0.002	0.01

a Genomic positions are according to NCBI build 36;

b Minor allele/major allele;

c The genotype counts are presented as homozygote/heterozygote/wildtype;

d Allelic *P* value has been adjusted for sex;

e Data from different study cohorts (initial cohort and replication cohort) were combined using Mantel-Haenszel models with fixed effects;

f P values after multiple correction (combined P*5).

We also tested the association of these 5 SNPs by using recessive and dominant models. Since 3 statistical models were used in the replication study, the p-value for significant observation should be 0.5/(5 SNP*3 models) = 0.0033. Only the SNP rs2075650 in the *TOMM40* had a significant association with human longevity of dominant model (*P* = 0.0000398, OR = 0.63 (0.51–0.79), **Table 4**). Another two SNPs (rs12978931 and rs395908) in the *PVRL2* gene showed a trend of association with human longevity of recessive model (*P* = 0.015 and 0.012, respectively; OR = 1.26 (95% CI 1.05–1.51) and 2.27 (95% CI 1.18–4.38), respectively; **Table 4**).

**Table 4:** Genotype Analysis of 5 SNPs by recessive and dominant models.

Gene	SNP	Position	Genotype	Case n(%)	Control n(%)	*P_rec* *	OR (95% CI)**	*P_dom* *	OR (95% CI)**
*PVRL2*	rs12978931	50055540	GG	256	367	0.015	1.26(1.05, 1.51)	0.444	1.07(0.90, 1.27)
			GA	480	866				
			AA	271	486				
*PVRL2*	rs519825	50058619	GG	24	26	0.103	1.59(0.91, 2.78)	0.001	1.34(1.12, 1.61)
			GA	246	342				
			AA	741	1355				
*PVRL2*	rs395908	50065405	CC	21	16	0.012	2.27(1.18, 4.38)	0.152	1.15(0.95, 1.39)
			CT	200	323				
			TT	785	1384				
*TOMM40*	rs2075650	50087459	AA	7	16	0.513	0.74 (0.30, 1.81)	3.98E-05	0.63 (0.51, 0.79)
			AG	123	310				
			GG	882	1398				
*APOE*	rs405509	50100676	CC	107	175	0.275	1.16 (0.89, 1.48)	0.028	1.19 (1.02, 1.39)
			CA	473	822				
			AA	428	875				

*P_rec, the *P* value of recessive model, *P_dom*, the P value of dominant model;

**OR, the odds ratio of homozygote; CI, Confidence Interval.

### Conditional, Disequilibrium and Haplotype Association Analysis of *PVRL2-TOMM40-APOE* locus

To verify our results, we took the three significant association SNPs (*PVRL2* rs519825, *TOMM40* rs2075650 and *APOE* rs405509) and sequentially conditioned on the minor allele of each variant. Results of the conditional analysis showed that rs2075650 was significant associated SNP with longevity in the *PVRL2-TOMM40-APOE* locus (**Table 5**). The analysis of linkage disequilibrium (LD) and haplotype block structure showed these SNPs were in the same LD block with each other (**Figure 1B**). Risk haplotype AACGA generated from these five SNPs proved to be significantly different between the cases and controls (*P* = 3.75×10^−9^, **Table 6**).

**Table 5:** Conditional analysis of the *PVRL2-TOMM40-APOE* locus in a replication cohort.

SNP ID	Position (bp)^a^	Gene	Allele^b^	Conditional on *PVRL2* rs519825^c^		Conditional on *TOMM40* rs2075650^c^		Conditional on *APOE* rs405509^c^	
				*P*	OR	*P*	OR	*P*	OR
rs12978931	50055540	*PVRL2*	G/A	0.80	0.97(0.77–1.21)	0.14	0.77(0.62–0.95)	0.50	0.88(0.63–1.25)
rs519825	50058619	*PVRL2*	G/A	-	-	0.77	1.04(0.77–1.40)	6.95E-04	
rs395908	50065405	*PVRL2*	T/C	0.68	1.07(0.77–1.47)	0.39	1.12(0.84–1.23)	0.98	0.99(0.7–1.4)
rs2075650	50087459	*TOMM40*	G/A	0.0000156	2.41(1.60–3.64)	-	-	7.84E-06	2.60(1.69–4.0)
rs405509	50100676	*APOE*	C/A	0.15	1.21(0.93–1.58)	0.79	0.97(0.78–1.20)	-	-

a Genomic positions are according to NCBI build 36;

b Minor allele/major allele;

c The results of association testing of the *PVRL2-TOMM40-APOE* locus when the allelic dosage of rs519825, rs2075650 or rs405509 was included in the regression model.

**Table 6:** The haplotype association with human longevity in the replication cohort at *PVRL2-TOMM40-APOE* locus.

Haplotype^a^	Frequency	Case, Control Frequencies	Chi Square	P Value
H1:AATGA	0.394	0.403, 0.384	0.691	0.4058
H2:GATGC	0.178	0.179, 0.177	0.018	0.8927
H3:GATGA	0.178	0.189, 0.165	1.684	0.1944
H4:GGTGC	0.116	0.107, 0.126	1.559	0.2118
H5:AACAA	0.077	0.101, 0.050	15.62	7.74×10^−5^
H6:AACGA	0.028	0.006, 0.053	34.846	3.57×10^−9^
H7:AATGC	0.014	0.009, 0.019	2.945	0.0861

a The haplotypes were generated from SNPs rs12978931, rs519825, rs395908, rs2075650 and rs405509.

## Discussion

In the present study, we investigated genetic variations contributing to longevity and observed that rs519825 in *PVRL2*, a 45 kb-distance with the neighbouring gene *APOE*, is associated with human longevity. These three SNPs, including rs2075650 in *TOMM40* and rs405509 in *APOE*, are located in at chromosome 19q13.32 in the *APOE* locus (**Figure 1**), which has shown consistent evidence for the association with longevity [1], [21], [23], [25]. The strength of this study is that, by case-control study, we have replicated the previously reported association of the *APOE* locus with longevity [1], [25] as the major locus. In addition, we found that rs519825 in *PVRL2*, around *TOMM40* and *APOE*, and rs405509 in *APOE* were associated with human longevity in the Han Chinese population.

To date, there is abundant evidence that human longevity is heritable based on twin studies and family studies, and approximately 25% of the overall variation in human lifespan can be attributed to genetic factors [3]–[5], which become more relevant for extreme longevity [6]. The Insulin/IGF-1 pathway has been reported to regulate lifespan extension in organisms ranging from invertebrates to mammals [29]. However, no convincing causal genes have yet been identified and efforts to map loci responsible for variation in human lifespan have limited success. One approach to investigate aging and longevity is to compare frequencies of genetic variants between nonagenarians or centenarians and the general population. In humans, both candidate gene and genome-wide genetic association approaches have been applied in an attempt to identify longevity loci. Multiple genes could mediate the aging process but would have their effects through numerous different patho-physiological processes and diseases that act as intermediate factors on the pathway to death [30], [31]. Therefore, any common variation in genes associated with aging probably has a small effect.

The more consistent evidence obtained by repeated observation in independent cohort studies for association with longevity has so far only been observed for three loci, the *APOE* locus [1], [25], [32], the *FOXO3A* locus [33]–[36] and the *AKT1* locus [34]. *APOE* is involved in lipoprotein metabolism and believed to pose an effect on longevity [1]. The genetic origin of the three common variants of the human apolipoprotein E (apoE) protein, known as E2, E3 and E4, has been related to a number of age-related diseases, including Alzheimer disease, as well as to healthy aging and longevity. The APOE ε4 haplotype is by far the most validated genetic variation and has repeatedly been associated with human longevity [19]–[25], [37]. The best known longevity variant rs2075650 in *TOMM40/APOE* has been showed to be associated with human longevity, it reached irrefutable genome wide significance and replicated in the independent cohorts including German, Dutch and Danish cohorts [21], [23], [28]. Schupf *et al* found that the frequency and likelihood of carrying a G allele in rs2075650 of *TOMM40* was lower among offspring in a long life family study (LLFS) in US Caucasian, compared with the likelihood in married-in controls [38]. Therefore, replication of this association in other long-lived cohorts will be needed to elucidate these results.

In our study, we applied a three-stage approach to investigate genetic variations contributing to longevity in a Han Chinese population. We found that three variations in *PVRL2* showed significant association with human longevity in initial cohort. Furthermore, the association of rs519825 in *PVRL2* could be replicated in another cohort, as well as the rs2075650 in *TOMM40* and rs405509 in *APOE*. Haplotype analysis showed that the three SNPs (rs2075650 in *TOMM40*, rs405509 in *APOE* and rs519825 in *PVRL2*) were in the same LD block. Rs7412 and rs429358 are not significant associated with human longevity in Chinese population in this study, this finding was consistent with previous report shown rs429358 and rs7412 were not significantly different between Super-Seniors and controls [37]. The G allele in rs2075650 of *TOMM40* was lower in the long-live individuals than that in the younger controls, which is consistent with previous studies [21], [23], [28], [38]. SNP rs405509 is a A/C variation upstream of the *APOE* gene, which has been reported to be associated with human lifespan [1], [21], [23], [25]. Rs2075650 is close to and in linkage disequilibrium with the rs429358, the APOE ε4 allele, and the investigators suggested that rs2075650 might not have an independent effect on longevity [21], [23], [28]. In this study, there were lower APOEε4 (rs429358) allele frequencies and higher ApoE ε2 (rs7412) allele frequencies in older group compared with younger cohort. This result suggested that ApoE ε4 carriers have an increased risk for mortality, while ApoE ε2 carriers are protected from diseases, which is consistent with previous studies [25], [39]. However, Yu *et al*'s study [40] showed that rs429358 and rs7412 are not in high linkage disequilibrium with each other, which suggested that they are not necessarily expected to behave the consistent associations. The controversy regarding the association of rs429358 and rs7412 with longevity is possibly due to differences in the study design and population control selection between the studies. These differences of APOE genotype may caused by different ethnic group and influenced by other genetic and environmental factors. Therefore, further study with an increased sample size would be needed in order to conclusively test for variant associations in APOE.

PVRL2 encodes a single-pass type I membrane glycoprotein with two Ig-like C2-type domains and an Ig-like V-type domain. This protein is one of the plasma membrane components of adherents junctions. Expression of PVRL2 has been detected in many organs [41] including brain (http://genecards.ccbb.re.kr/cgi-bin/carddisp.pl?gene=PVRL2) and in several neuronal cell lines [42], [43]. So far, the biological relationship of PVRL2, as well as TOMM40 and APOE with human longevity, have not been well evaluated. Therefore, it is meaningful to explore the role of PVRL2 in influencing human longevity. Further biological studies will be helpful to explore the joint effects of different gene variants in longevity-related pathways and determine which genes are actually involved in the human longevity pathogenesis.

In conclusion, our results replicated the association between human longevity and a known variant in *TMM40* near *APOE* in the mainland Han Chinese population. In addition, we have found another two variants (rs519825 in *PVRL2* and rs405509 in *APOE*) were significantly associated with human longevity. The three SNPs are localized in *APOE* locus, which is universally recognized as a major disease susceptibility locus for human longevity, suggesting that this locus may contribute to human longevity in the Han Chinese population. Therefore, future exact studies in much larger cohorts to identify the causal variants and functional study are very important to verify whether any of these genes are involved in the development of human longevity.
